# First-void urine and cervicovaginal brushes for vaginal microbiome profiling: a proof-of-concept study

**DOI:** 10.1128/spectrum.00096-26

**Published:** 2026-08-19

**Authors:** Maria Pinedo-Bardales, Anne Van Caesbroeck, Sarah Ahannach, Margo Bell, Severien Van Keer, Laura Téblick, Isabel Erreygers, Sam Bakelants, Sarah Van den Bosch, Sandra Condori-Catachura, Stijn Wittouck, Alex Vorsters, Sarah Lebeer

**Affiliations:** 1 Laboratory of Applied Microbiology and Biotechnology, Department of Bioscience Engineering, University of Antwerp 692679, Antwerp, Belgium; 2 Centre for the Evaluation of Vaccination (CEV), Vaccine & Infectious Disease Institute (VAXINFECTIO), Faculty of Medicine and Health Sciences, University of Antwerp 26660, Antwerp, Belgium; 3 U-MaMi Centre of Excellence, University of Antwerp 26660, Antwerp, Belgium; Third Affiliated Hospital of Sun Yat-sen University, Guangzhou, China; Loyola University Chicago, Maywood, Illinois, USA

**Keywords:** vaginal microbiome, first-void urine, vaginal sample, self-sampling, women’s health

## Abstract

The human vaginal microbiome is a cornerstone of female reproductive health, influencing susceptibility to vaginal conditions, pregnancy complications, and urogenital infections. Understanding this microbiome is critical for advancing preventive, diagnostic, and therapeutic interventions, yet its intimate and dynamic nature poses challenges for accurate, representative, and more inclusive sampling methods. This proof-of-concept study evaluated the performance of first-void urine (FVU) samples and cervicovaginal brushes for vaginal microbiome profiling relative to vaginal swabs. FVU refers to the initial stream of urine that flushes away cervicovaginal secretions that accumulate between the small labia and around the urethral opening, capturing immune, cancer, disease, pathogen, and microbiome biomarkers. Next to FVU, we evaluated cervicovaginal brushes, which are commonly used as self-sampling devices for human papillomavirus (HPV) DNA screening and thus present an interesting opportunity for evaluating the cervicovaginal microbiome while also collecting human vaginal cells. Here, we performed microbiome profiling in 10 women based on a total of 40 samples. In six out of nine participants, high-quality microbial profiles were obtained for both fresh FVU and cervicovaginal brush samples, comparable to those derived from vaginal swabs. Storage at room temperature for 7 days influenced the microbial composition of four out of seven FVU samples. Overall, our findings demonstrate the potential of FVU as a practical and accessible self-sampling method for advancing women’s health research.

**IMPORTANCE:**

Current approaches for mapping and assessing the vaginal microbiome predominantly rely on sampling through vaginal swabs. However, their use can be perceived as uncomfortable, and cultural norms, stigmas, and privacy concerns in certain communities can also limit their acceptability and accessibility, especially in minors. In this proof-of-concept study, we show that first-void urine (FVU) samples and cervicovaginal brushes can be used as non-invasive, user-friendly alternatives in vaginal microbiome research, enabling more accessible and scalable methods for microbiome composition analysis, paving the way for large-scale applications in future projects. This would also enable us to conduct a longitudinal study of the vaginal microbiome in children and to explore its evolution through to adulthood, capturing microbial community dynamics that remain largely uncharacterized. Such improvements could strengthen our understanding of the vaginal microbiota and their role in women’s health.

## OBSERVATION

Ten self-reported healthy women of reproductive age were enrolled at the Center for the Evaluation of Vaccination in Antwerp (Belgium) in October 2023. Experimental methods are described in detail in the Supplemental Material. Briefly, participants were randomized into two groups differing in sampling order (A and B). Each participant self-collected four samples (Fig. S1): a first-void urine (FVU) sample, collected using the 20 mL Colli-Pee device (Novosanis, part of OraSure Technologies Inc. Group, USA), prefilled with a nucleic acid preservative [urine conservation medium (UCM), DNA Genotek Inc., Canada], two vaginal eNAT swabs (Copan, Italy) including 1 mL of nucleic acid preservation medium, and a cervicovaginal sample using the Evalyn Brush (Rovers Medical Devices B.V., the Netherlands), which was resuspended in 20 mL of ThinPrep PreservCyt solution (Hologic, USA). For each participant, one vaginal swab and one FVU aliquot were kept at room temperature (RT) for up to 7 days before freezing at −20°C to assess storage effects. All other aliquots were immediately frozen at −20°C. A storage temperature of −20°C was favored over −80°C to facilitate implementation for at-home sampling, while RT storage supports implementation in large-scale sampling campaigns with transport at RT (1). DNA extraction was then performed on 1 mL aliquots of each sample type.

**Fig 1:**
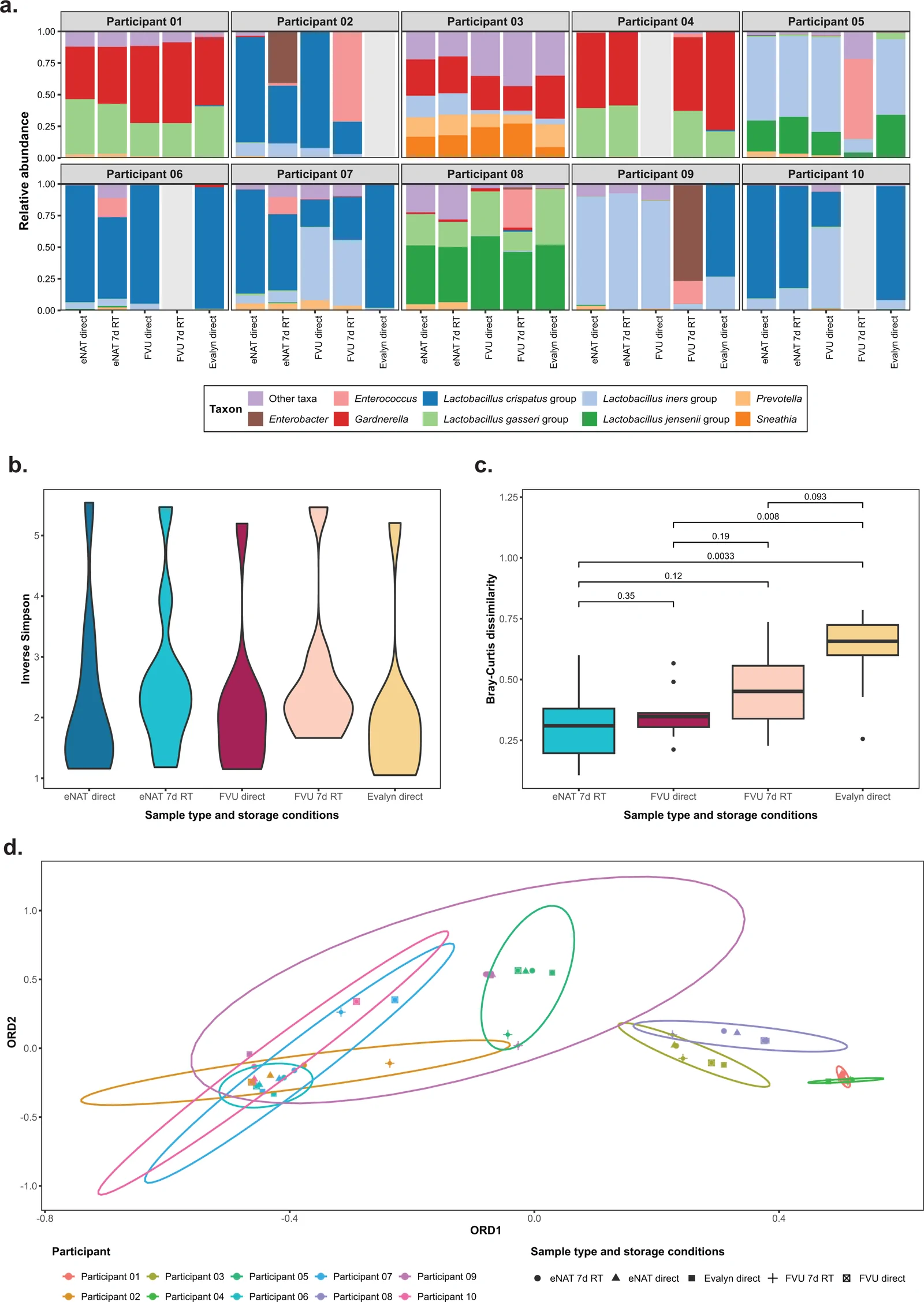
Comparison of sample collection methods and storage conditions in terms of (**a**) taxonomic composition, (**b**) alpha diversity (Inverse Simpson index), and (**c**) beta diversity (based on the presence/absence of taxa) relative to directly frozen vaginal swabs. Bray-Curtis (BC) dissimilarities between each sampling method of all participants, resulting from the eNAT direct (gold standard), grouped by sampling method to evaluate how different sampling methods could affect the detected microbiome composition. Statistics were performed with the Wilcoxon signed-rank test. Data are represented as mean ± SEM. A BC dissimilarity index of 0 indicates that the bacterial composition between the used methods is the same as a directly processed vaginal swab, whereas a beta-diversity of 1 indicates that the composition differs completely. The BC dissimilarity index was significantly higher in FVU samples stored at RT for 7 days compared to vaginal swabs stored at RT for 7 days and directly processed Evalyn brushes. Panel **d** displays the Principal Coordinates Analysis (PCoA), with each participant indicated by a distinct color. Relative abundances could not be determined in samples where high-quality reads were lacking, which is indicated by a gray bar. Taxonomic profiles of high-quality samples and diversity metrics were visualized using the in-house developed R package tidytacos. The Wilcoxon signed-rank test was used to determine statistical significance.

After DNA extraction, the V4 region of the 16S rRNA gene was amplified and sequenced using dual-index paired-end sequencing (2), with libraries prepared following standard in-house protocols (1) and controls included (Fig. S2). Amplicon reads were processed using the R package DADA2 (3) version 1.22.0 as described before (4). Resulting amplicon sequence variants (ASVs) were taxonomically assigned from phylum to genus level using the Genome Taxonomy Database (GTDB) (5). *Lactobacillus* ASVs were reclassified into nine previously validated subgenera (3), while *Bifidobacterium* ASVs were reclassified using the SILVA database (6) (v138). An overview of the quality of samples and sequencing metrics is summarized in Table S1. Taxonomic profiles were visualized using the tidytacos package (5) (Fig. 1a). Alpha diversity was assessed using the inverse Simpson index (Fig. 1b), and beta diversity using the Bray-Curtis (BC) dissimilarity index. Additional analyses included the calculation of BC dissimilarity indices based on binarized data (taxa presence/absence, Fig. 1c) and Principal Coordinates Analysis (PCoA) for clustering (Fig. 1d). Statistical comparisons between sampling methods were performed using the Wilcoxon signed-rank test with Holm-Bonferroni correction.

After quality control, vaginal swabs stored at RT for 7 days yielded the highest mean read concentration (17,841 reads/µL), followed by directly frozen vaginal swabs (13,372 reads/µL), FVU samples stored at RT for 7 days (9,292 reads/µL), directly frozen FVU (7,647 reads/µL), and cervicovaginal brushes (1,830 reads/µL) (Fig. S2; Table S1). Of note, in vaginal microbiome research, substantial inter-individual variation is commonly observed (7), which may outweigh differences attributable to sampling method. High-quality reads were obtained from all vaginal swabs and most directly frozen FVU samples and cervicovaginal brush samples (9 out of 10 samples each). Two FVU samples stored at RT failed quality control, possibly due to suboptimal bacterial DNA preservation under these storage conditions, the inherently low microbial biomass collected by some sampling devices (8, 9), or differences in the volume of preservation medium used by each device. Overall, taxonomic profiles were successfully obtained for all sampling methods and protocols tested in six participants (participants 01, 03, 05, 07, 08, and 09; Fig. 1a).

When assessing directly frozen samples, eight vaginal swabs were predominated (taxa with a relative abundance of 30% or above) by *Lactobacillus* species (Fig. 1a; Table S1), including species from the *Lactobacillus crispatus* group (participants 02, 06, 07, and 10), *Lactobacillus iners* group (participants 05 and 09), and the *Lactobacillus jensenii* group (participant 08). Participants 01 and 04 were predominated by a combination of *Lactobacillus gasseri* group and *Gardnerella*. Participant 03 exhibited a more diverse profile, mostly comprised of *L. iners* group, *Gardnerella, Sneathia, and Prevotella*. Directly frozen FVU samples and cervicovaginal brushes generally shared the same predominant taxon with the corresponding directly frozen vaginal swabs (eight out of nine participants), with exceptions for participant 04 (FVU) and participant 09 (cervicovaginal brushes).

When vaginal swabs were stored at RT for 7 days, the taxonomic composition remained stable when compared to their respective freshly stored swab, except for participant 02 (Fig. 1a; Table S1). In FVU samples, only four out of seven participants with high-quality samples for both protocols had the same predominant taxon after RT storage (participants 01, 03, 07, and 08). Notably, storage led to an increased presence of usually less relatively abundant vaginal taxa, such as *Enterococcus* and/or *Enterobacter*, possibly reflecting microbial shifts under suboptimal storage conditions or overgrowth of facultative anaerobes (potentially originating from fecal or skin bacteria) at RT. Consistently, higher dissimilarity was observed in FVU samples stored at RT (based on both relative abundance and presence/absence analyses) compared to paired vaginal swabs. BC dissimilarity was significantly higher in FVU samples kept at RT for 7 days compared to eNAT swabs kept at RT for 7 days and directly processed cervicovaginal brush samples. Other differences between individual sampling protocols were non-significant (Fig. 1c). However, these differences may partly reflect within-individual variation rather than true differences between sampling methods. To explore this, we performed a PCoA, which showed that sample types and protocols primarily clustered by participant (Fig. 1d).

**Fig 2:**
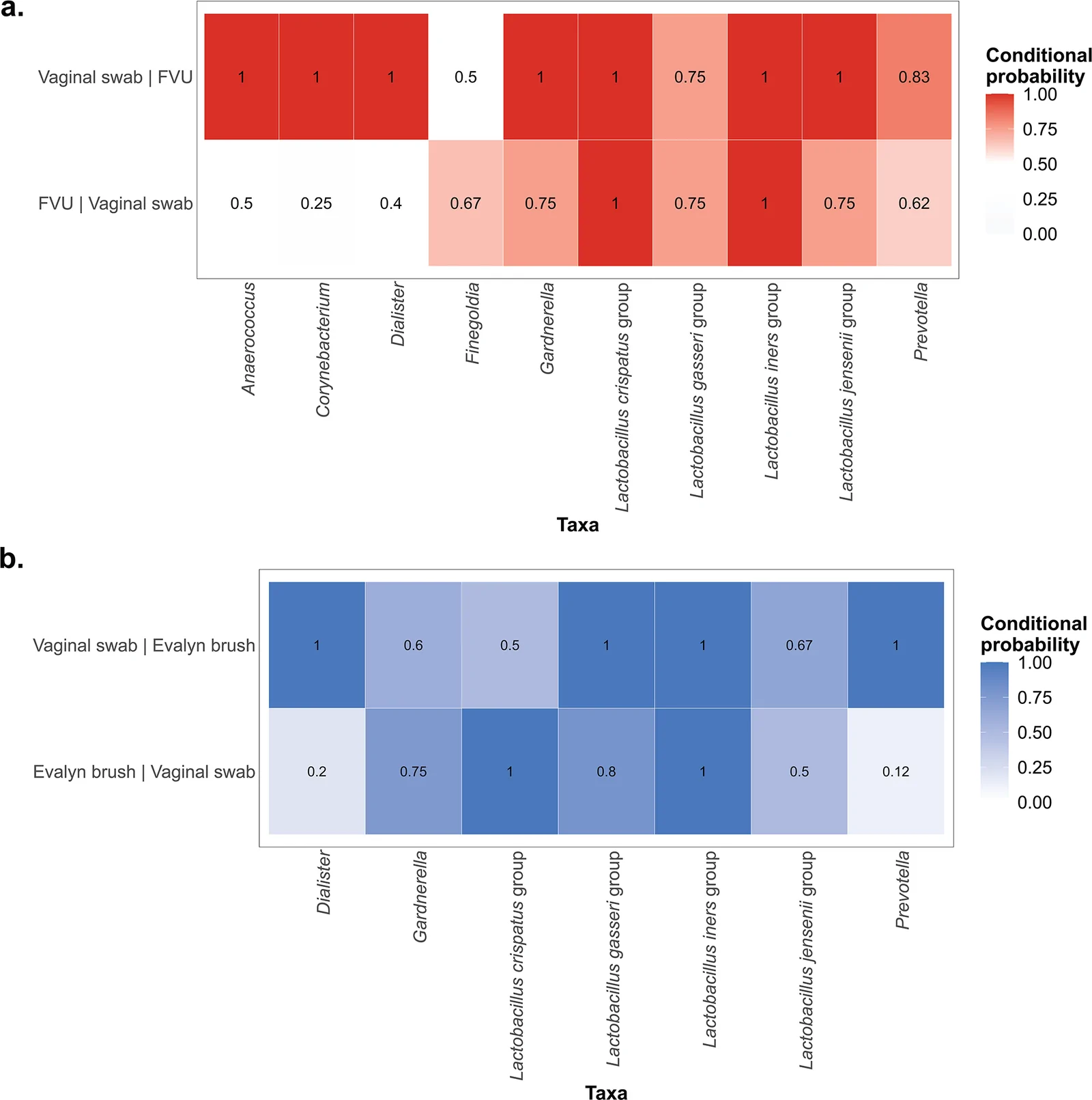
Taxon-level concordance between directly frozen vaginal swabs and alternative sample types. Heatmaps showing conditional probabilities of taxon detection between directly frozen vaginal swabs (eNAT; reference standard) and directly frozen (**a**) first-void urine (FVU) samples, and (**b**) cervicovaginal brush samples. Each tile represents the probability of detecting a given taxon in one sample type given its presence in the other, as indicated on the y-axis. Values (range 0–1) are displayed within tiles. Color intensity reflects increasing probability (white to red for FVU; white to blue for cervicovaginal brush samples). Only taxa with relative abundance ≥0.5% and detected in at least five samples across both sample types were included.

Next, the taxon-level concordance between directly frozen samples was assessed using presence/absence data derived from relative abundances (threshold ≥0.5%). Conditional probabilities were calculated to evaluate the likelihood of detecting each taxon in alternative sample types given its presence in the vaginal swabs (10). Concordance between vaginal swabs and FVU samples was high for predominant *Lactobacillus* species, mainly for *L. iners* group and *L. crispatus* group, which showed near-perfect agreement (Fig. 2a). Similarly, strong concordance was observed between vaginal swabs and cervicovaginal brush (Evalyn) samples, especially for *L. iners* group and *L. gasseri* group (Fig. 2b). In contrast, non-*Lactobacillus* taxa such as *Dialister* and *Prevotella* demonstrated lower concordance, with substantially reduced probabilities of detection in FVU and cervicovaginal brush samples compared to vaginal swabs. Notably, detection in FVU or cervicovaginal brush samples was generally accompanied by detection in the vaginal samples, whereas the reverse was less consistent. While predominant vaginal taxa are reliably detected across sample types, concordance decreases for less abundant and more variable taxa.

Overall, the consistent taxonomic similarity between the profiles from vaginal swabs, cervicovaginal brushes, and directly frozen FVU samples highlights their potential for vaginal microbiome research. Of note, PCoA showed that samples clustered mainly by participant, suggesting that microbial profiles are largely participant-specific rather than driven solely by sampling method. Despite our relevant findings, the small sample size limits robust comparisons, and differences in sample collection, storage, and handling may introduce biases. Interestingly, the quality of the samples is linked to the device used for sample collection, limiting extrapolation to other sampling devices. In addition, RT storage of cervicovaginal brushes was not assessed due to practical and economic considerations. Moreover, it would be of interest to collect paired midstream urine samples in a future investigation, which will help determine whether bacteria found in FVU are vagina-derived, bladder-derived, or a composite of both. The study design prioritized within-subject comparisons, precluding conclusions on sampling order effects. Furthermore, the use of 16S rRNA sequencing, instead of shotgun metagenomics, limits taxonomic resolution, although it remains a cost-effective and widely used method, and our amplicon sequencing pipeline for *Lactobacillus* (sub)genus-level classification has been rigorously validated in a previous study (4). Altogether, FVU samples represent a practical proxy for vaginal microbiome profiling. This can be especially advantageous in large-scale or remote studies, or in projects including hard-to-reach populations such as girls before sexual debut or women uncomfortable with vaginal self-sampling. Ease of sample collection is especially important from a citizen-science perspective to reduce participation barriers and increase inclusion of underserved populations (11). In this regard, FVU is non-invasive and easier to collect compared to vaginal swabs or cervicovaginal brushes, making it a promising sample type for future microbiome research.

## Supplementary Material

Reviewer comments

## Data Availability

Sequencing data from high-quality samples included in this study are available at the European Nucleotide Archive under bioproject PRJEB87599. Samples were stored at Biobank Antwerp (Belgium, ID: BE 71030031000) (12).

## SUPPLEMENTAL MATERIAL

Fig. S1 spectrum.00096-26-s0001.tiff Overview of the study workflow.

Fig. S2 spectrum.00096-26-s0002.tiff Quality control of the samples based on the negative controls.

Supplemental materials spectrum.00096-26-s0003.docx Supplemental text, legends for Fig. S1 and S2, and Table S1.

OPEN PEER REVIEW reviewer-comments.pdf An accounting of the reviewer comments and feedback.

ASM does not own the copyrights to Supplemental Material that may be linked to, or accessed through, an article. The authors have granted ASM a non-exclusive, world-wide license to publish the Supplemental Material files. Please contact the corresponding author directly for reuse.

## References

[1] Ahannach S, Delanghe L, Spacova I, Wittouck S, Van Beeck W, De Boeck I, Lebeer S. 2021. Microbial enrichment and storage for metagenomics of vaginal, skin, and saliva samples. iScience 24:103306. doi:10.1016/j.isci.2021.103306 PMC8571498 34765924

[2] Kozich JJ, Westcott SL, Baxter NT, Highlander SK, Schloss PD. 2013. Development of a dual-index sequencing strategy and curation pipeline for analyzing amplicon sequence data on the MiSeq Illumina sequencing platform. Appl Environ Microbiol 79:5112–5120. doi:10.1128/AEM.01043-13 PMC3753973 23793624

[3] Callahan BJ, McMurdie PJ, Rosen MJ, Han AW, Johnson AJA, Holmes SP. 2016. DADA2: high-resolution sample inference from Illumina amplicon data. Nat Methods 13:581–583. doi:10.1038/nmeth.3869 PMC4927377 27214047

[4] Lebeer S, Ahannach S, Gehrmann T, Wittouck S, Eilers T, Oerlemans E, Condori S, Dillen J, Spacova I, Vander Donck L, Masquillier C, Allonsius CN, Bron PA, Van Beeck W, De Backer C, Donders G, Verhoeven V. 2023. A citizen-science-enabled catalogue of the vaginal microbiome and associated factors. Nat Microbiol 8:2183–2195. doi:10.1038/s41564-023-01500-0 PMC10627828 37884815

[5] Wittouck S, Rillaer TV, Smets W, Lebeer S. 2025. Tidytacos: an R package for analyses on taxonomic composition of microbial communities. J Open Source Softw 10:6313. doi:10.21105/joss.06313

[6] Quast C, Pruesse E, Yilmaz P, Gerken J, Schweer T, Yarza P, Peplies J, Glöckner FO. 2013. The SILVA ribosomal RNA gene database project: improved data processing and web-based tools. Nucleic Acids Res 41:D590–D596. doi:10.1093/nar/gks1219 PMC3531112 23193283

[7] Ravel J, Gajer P, Abdo Z, Schneider GM, Koenig SSK, McCulle SL, Karlebach S, Gorle R, Russell J, Tacket CO, Brotman RM, Davis CC, Ault K, Peralta L, Forney LJ. 2011. Vaginal microbiome of reproductive-age women. Proc Natl Acad Sci USA 108:4680–4687. doi:10.1073/pnas.1002611107 PMC3063603 20534435

[8] Greathouse KL, Sinha R, Vogtmann E. 2019. DNA extraction for human microbiome studies: the issue of standardization. Genome Biol 20:212. doi:10.1186/s13059-019-1843-8 PMC6802309 31639026

[9] Kumar T, Bryant M, Cantrell K, Song SJ, McDonald D, Tubb HM, Farmer S, Lewis A, Lukacz ES, Brubaker L, Knight R. 2024. Effects of variation in sample storage conditions and swab order on 16S vaginal microbiome analyses. Microbiol Spectr 12:e03712-23. doi:10.1128/spectrum.03712-23 PMC10783137 38095462

[10] Sung J, Larsen P, Halverson TM, Waters TP, Goodman JR, Wolfe AJ. 2024. First trimester “clean catch” urine and vaginal swab sample distinct microbiological niches. Microbiol Spectr 12:e0263823. doi:10.1128/spectrum.02638-23 PMC10782990 38088549

[11] Ahannach S, Van Hoyweghen I, Verhoeven V, Lebeer S. 2024. Citizen science as an instrument for women’s health research. Nat Med 30:3445–3454. doi:10.1038/s41591-024-03371-2 39578585

[12] BE 71030031000; Biobank Antwerp [BB190007]. 2021. [BIORESOURCE]. BBMR-ERIC Belgian

