# Supplementary material for: First-void urine and cervicovaginal brushes for vaginal microbiome profiling: a proof-of-concept study
Source: Microbiol Spectr. 2026 Aug 19;14(10):e00096-26. doi: 10.1128/spectrum.00096-26 (PMC13689475; doi:10.1128/spectrum.00096-26)
Supplement: Supplementary file 1 [file reviewer-comments.pdf]

ASM | Microbiology Spectrum

First-void urine and cervicovaginal brushes for vaginal microbiome profiling: a proof-of-concept study

Maria Pinedo-Bardales, Anne Van Caesbroeck, Sarah Ahannach, Margo Bell, Severien Van Keer, Laura Téblick, Isabel Erreygers, Sam Bakelants, Sarah Van den Bosch, Sandra Condori-Catachura, Stijn Wittouck, Alex Vorsters, and Sarah Lebeer

Corresponding Author(s): Sarah Lebeer, Universiteit Antwerpen

Review Timeline:

Submission Date:	February 18, 2026
Editorial Decision:	April 28, 2026
Revision Received:	June 26, 2026
Accepted:	July 17, 2026

Editor: Zhe Li

Reviewer(s): Disclosure of reviewer identity is with reference to reviewer comments included in decision letter(s). The following individuals involved in review of your submission have agreed to reveal their identity: Alan J Wolfe (Reviewer #1)

Transaction Report:

(Note: With the exception of the correction of typographical or spelling errors that could be a source of ambiguity, letters and reports are not edited. The original formatting of letters and referee reports may not be reflected in this compilation.)

DOI: https://doi.org/10.1128/spectrum.00096-26

Re: Spectrum00096-26 (**First-void urine and cervicovaginal brushes for vaginal microbiome profiling: a proof-of-concept study**)

Dear Prof. Sarah Lebeer:

Thank you for the privilege of reviewing your work. Below you will find my comments, instructions from the Spectrum editorial office, and the reviewer comments.

Please return the manuscript within 60 days; if you cannot complete the modification within this time period, please contact me. If you do not wish to modify the manuscript and prefer to submit it to another journal, notify me immediately so that the manuscript may be formally withdrawn from consideration by Spectrum.

Revision Guidelines
To submit your modified manuscript, log into the submission site at https://spectrum.msubmit.net/cgi-bin/main.plex. Go to Author Tasks and click the appropriate manuscript title to begin. The information you entered when you first submitted the paper will be displayed; update this as necessary. Note the following requirements:

• Upload point-by-point responses to the issues raised by the reviewers in a file named "Response to Reviewers," NOT in your cover letter.
• Upload a compare copy of the manuscript (without figures) as a "Marked-Up Manuscript" file.
• Upload a clean .DOC/.DOCX version of the revised manuscript and remove the previous version.
• Each figure must be uploaded as a separate, editable, high-resolution file (TIFF or EPS preferred), and any multipanel figures must be assembled into one file.
• Any supplemental material intended for posting by ASM should be uploaded with their legends separate from the main manuscript. You can combine all supplemental material into one file (preferred) or split it into a maximum of 10 files with all associated legends included.

For complete guidelines on revision requirements, see our Submission and Review Process webpage. Submission of a paper that does not conform to guidelines may delay acceptance of your manuscript.

Data availability: ASM policy requires that data be available to the public upon online posting of the article, so please verify all links to sequence records, if present, and make sure that each number retrieves the full record of the data. If a new accession number is not linked or a link is broken, provide Spectrum production staff with the correct URL for the record. If the accession numbers for new data are not publicly accessible before the expected online posting of the article, publication may be delayed; please contact production staff (Spectrum@asmusa.org) immediately with the expected release date.

Publication Fees: For information on publication fees and which article types are subject to charges, visit our website. If your manuscript is accepted for publication and any fees apply, you will be contacted separately about payment during the production process; please follow the instructions in that e-mail. Arrangements for payment must be made before your article is published.

ASM Membership: Corresponding authors may join or renew ASM membership to obtain discounts on publication fees. Need to upgrade your membership level? Please contact Customer Service at Service@asmusa.org.

The ASM Journals program strives for constant improvement in our submission and publication process. Please tell us how we can improve your experience by taking this quick Author Survey.

Thank you for submitting your paper to Spectrum.

Sincerely,
Zhe Li
Editor
Microbiology Spectrum

Reviewer #1 (Comments for the Author):

In this resubmission, the authors have responded positively to many of the reviewers' concerns, including several of mine. But I still have concerns, some related to methodology and some to interpretation.

Methodology

Line 87: The authors did not use a nucleic preservative. And they froze the samples at -20C instead of -80C. I understand the reasoning for freezing at -20C, and the authors SHOULD explain this reasoning. I'm not sure why they didn't use a nucleic acid preservative. It is possible to provide a preservative that participants can add to their sample or add their sample to the preservative. Others have done so and it allows preservation at RT for up to a week prior to freezing.

Line 108: The authors state that "additional analyses included the calculation of BCs based on taxa presence/absence." The Bray-Curtis Dissimilarity index is quantitative, accounting for taxon abundance. In contrast, the Jaccard Dissimilarity index is qualitative, focusing strictly on the presence or absence of species. So, I don't understand this statement. Do the authors mean that they used the Jaccard index instead? Please explain.

Interpretation:
Line 109: The beta diversity analysis shown in panel D mostly shows that the participants differ quite a lot, so much so that attempts to compare sample types from the different participants can be very biased by individual participants. Thus, it does not mean what the authors claim it means.

Line 142: Why would the FVU samples of groups A and B differ so much? Group A first collected FVU followed by the collection of two vaginal eNAT swabs..., whereas Group B first collected FVU followed by the cervicovaginal brush...." If the FVUs were collected first by both groups, then they should be somewhat similar. They are not at all. I have an extremely likely explanation: the participants differ significantly from each other so combining their samples and comparing two groups is biased by the accident of the randomness of participant assignment to those groups.

Line 158: Whilst the authors acknowledge some of the limitations of their study, I am convinced that they do not go far enough. First, I understand why they used short read 16S sequencing but they do not acknowledge that it limits identification with confidence at the species level even if they query some of their sequence reads against additional databases - there is just not sufficient sequence data for distinction with confidence between many closely related species. Furthermore, there's no mention of confidence scores for the taxonomic identifications. How confident are the authors that their identifications are accurate? This is important for the second issue. At the level of their analysis, the authors cannot be certain that the FVU samples truly resemble the vaginal swabs below the level of the most relatively abundant taxa. I stand by my original concern (reproduced below).

"Third, and importantly, the authors base their conclusions on whether the sample composition in terms of alpha (within sample) diversity and/or the identity of the predominant taxon differed. In general, I agree that a simple glance at the data suggests that there is not much difference between the 3 different types of samples for most participants. However, the authors do not determine the likelihood that a taxon detected in the vaginal swabs will also be detected in the FVU or cervicovaginal brush samples. If one only looks at the most relatively abundant taxa, they may miss that important information may be lost by using a proxy sample, such as FVU. The authors should take a look at a recent publication comparing midstream voided urine and vaginal swabs in pregnant females (Sung et al., 2024 PMID: 38088549). These authors asked that very question and found that the two sample types were not particularly good proxies for each other. The authors could use the analytical methods used in this paper to determine whether FVU samples are good proxies for vaginal swabs in this population."

Now I understand what the authors are trying to do, and I believe that it is a worthy endeavor, but they MUST include this concern as a limitation. They MUST tell their readers how this simple, non-invasive approach will limit interpretation of the resultant data. Furthermore, the samples represent the urogenital tract and may not be a great proxy for what is actually in the vagina itself. The results will NOT be vaginal microbiome but rather a composite of the entire urogenital microbiome.

Other comments
Line 126: Why was this more interesting than the other results? Delete the "interesting." It does nothing positive for the logic flow. Note that these types of transition words usually hold no value as the reader has no idea why an observation was "interesting," "surprising," or "intriguing." If it was interesting, surprising, or intriguing, answer the question "why?" and then use your answer as an transition/introductory phrase.

Line 127: "...were predominated by a combination of ..." is a better more accurate way to say what the authors mean.
Line 138: More accurate is "...less relatively abundant..." because 16S sequencing is semi-quantitative due to several different biases inherent in the method that affect quantification.

Line 142: Remind the reader how the groups differ. They are described at the very beginning of the Observation, and I had forgotten by the time I got to this sentence.

Line 153: Yes. Biases. But the differences between the participants means that you cannot compare them. They have to be their own controls.

Reviewer #2 (Comments for the Author):

Revised manuscript is significantly changed from its original submission. The major change is complete deletion of the qPCR data related to estimating human and bacterial DNA and analyses of that data. This is a pity as such data would have strengthened this pilot study. Authors refer to some sort of 'inconsistencies' in those measurements in their response to several reviewers but it did not provide too much clarify on the issue identified. Unfortunately, the physical samples are stated to be used up and no longer available for re-analyses.

>> The revised manuscript now has the PCoA analyses for patients and groups.
The response file contains the third PCoA analysis for sample storage protocol (Direct freezing or after 7 d RT) - It would be great to include that to the supplemental materials for this manuscript.

>> Response file/Abstract/Results and Figure 2 legends differ in interpretation of the presented data on sampling order.
Figure 2 legend states that PCoA is nonsignificant for both plots given (by participant and by group) while Abstract/Response file claim clear clusters and differences.

>> Supplementary Table is not listed in Manuscript's list of Supplementary materials. This table also lacks units or descriptions of values (for example, DNA concentration or Read concentration lack units). Samples in the tables are not sorted for some reason by patient or other cohorts and it is not immediately clear why some participants only have 4 samples instead of 5 (three collection modes with two samples being left out at RT for 7 days).

>> Observation format is limiting the word numbers for this manuscript, and thus is perhaps prohibitive to inclusion of all the details added in the reviewers comments. Given the importance of the experimental and protocol details for this kind of studies I would suggest including those details into the supplemental materials to avoid any confusions for future readers.

>> Additionally, authors did not include any mentioning or acknowledging the urine microbiome or urogenital microbiome as similar or distinct to the vaginal microbiome. It is interesting choice and in particular in regard to recent study mentioned by another reviewer (Sung et al, 2023). Authors refer to the studies from HPV field which seems to be more advanced in developing protocols for viral DNA detection and sampling methods for a few research years. Nevertheless, the human-cell specific virus might not necessarily be a good standard as authors aim to analyze composition of bacteria in those microbial niches and stability of cells and DNA, accumulation of host or microbial cells and so one - might differ. This actually stresses the need for this pilor study but calls for attention to details in experimental descriptions and interpretation of the results.

Reviewer's Comments and Authors' Response

Manuscript number: Spectrum00096-26

Manuscript title: First-void urine and cervicovaginal brushes for vaginal microbiome profiling: a proof-of-concept study.

Authors: Maria Pinedo-Bardales*, Anne Van Caesbroeck*, Sarah Ahannach, Margo Bell, Severien Van Keer, Laura Téblick, Isabel Erreygers, Sam Bakelants, Sarah Van den Bosch, Sandra Condori-Catachura, Stijn Wittouck, Alex Vorsters°, Sarah Lebeer°. (*shared first authors; °shared senior authors)

The authors would like to thank the reviewers for their time spent carefully reviewing the manuscript and for their valuable comments and suggestions. We have addressed all of them in the responses below. The corresponding changes and refinements made in the revised manuscript are summarized in our responses. In what follows, the reviewers' comments are in black and the authors' responses are in blue.

<u>Reviewer #1 (Comments for the Author)</u>:

In this resubmission, the authors have responded positively to many of the reviewers' concerns, including several of mine. But I still have concerns, some related to methodology and some to interpretation.

1. Methodology: Line 87: The authors did not use a nucleic preservative. And they froze the samples at -20C instead of -80C. I understand the reasoning for freezing at -20C, and the authors SHOULD explain this reasoning. I'm not sure why they didn't use a nucleic acid preservative. It is possible to provide a preservative that participants can add to their sample or add their sample to the preservative. Others have done so and it allows preservation at RT for up to a week prior to freezing.

Answer: We thank the reviewer for this comment. Indeed, nucleic preservatives and storage temperature are important considerations. First-void urine (FVU) samples were collected in Urine Conservation Medium (UCM). This buffer contains a nucleic acid preservative, which has now been clarified in the revised manuscript in lines 77 – 79. All samples were indeed stored at -20°C rather than -80°C, in accordance with the optimized protocol for DNA extraction and microbiome profiling starting from eNAT swabs as described in the methodological optimization preceding the large-scale citizen-science study Isala (Ahannach S et al., iScience 2021). Using this storage approach ensures comparability with previously collected samples and facilitates potential future implementation for at-home sample collection and storage. This clarification has been incorporated into the revised manuscript (lines 93 – 96).

Of note, in addition to the samples analysed in this manuscript, we also evaluated an extra FVU aliquot (collected in UCM) to which eNAT buffer (Copan) was added in a 1:1 ratio. The eNAT buffer is a guanidine thiocyanate-based solution that stabilizes and preserves nucleic acids. These samples were stored at room temperature (RT) for one week prior to freezing. However, this approach resulted in lower DNA concentrations due to sample dilution, and consequently, these samples failed quality control across all participants and were excluded from the final analysis presented in the manuscript.

2. Line 108: The authors state that "additional analyses included the calculation of BCs based on taxa presence/absence." The Bray-Curtis Dissimilarity index is quantitative, accounting for taxon abundance. In contrast, the Jaccard Dissimilarity index is qualitative, focusing strictly on the presence or absence of species. So, I don't understand this statement. Do the authors mean that they used the Jaccard index instead? Please explain.

Answer: We thank the reviewer for this important observation and agree that Bray-Curtis dissimilarity is fundamentally an abundance-based metric, whereas presence/absence data are classically summarized using indices such as the Jaccard dissimilarity index. In our analysis, we used the *vegan* package implementation of Bray-Curtis dissimilarity within the *tidytacos* workflow, which allows calculation on both quantitative (abundance) data and binary (presence/absence) data. Specifically, for the presence/absence-based analyses, the count table was first transformed to a binary format prior to distance calculation. When Bray-Curtis is applied to such binarized data, it effectively represents a qualitative dissimilarity measure that reflects shared versus non-shared taxa and is mathematically equivalent to the Sørensen (Dice) index rather than the Jaccard index. We acknowledge that our

original wording may have been unclear. To avoid confusion, we have revised the manuscript to explicitly state that Bray-Curtis dissimilarity was calculated on binarized data, and to clarify its interpretation as a presence/absence-based metric (line 120), full explanation in the Supplementary file). In addition, the correct figure corresponding to these analyses has been added as Figure 1c.

3. Interpretation: Line 109: The beta diversity analysis shown in panel D mostly shows that the participants differ quite a lot, so much so that attempts to compare sample types from the different participants can be very biased by individual participants. Thus, it does not mean what the authors claim it means.

Answer: We thank the reviewer for this important observation. We agree that the beta diversity analysis (Figure 1d) reveals substantial inter-individual variation, with samples clustering primarily by participant rather than by sampling method. This is consistent with established findings in vaginal microbiome research, where participant-specific community composition often represents a major source of variation. To address this point, we have revised the text (lines 131 – 133) to clarify that differences between sampling methods should be interpreted with caution, as they may be influenced by participant-specific microbiome profiles.

4. Line 142: Why would the FVU samples of groups A and B differ so much? Group A first collected FVU followed by the collection of two vaginal eNAT swabs..., whereas Group B first collected FVU followed by the cervicovaginal brush...." If the FVUs were collected first by both groups, then they should be somewhat similar. They are not at all. I have an extremely likely explanation: the participants differ significantly from each other so combining their samples and comparing two groups is biased by the accident of the randomness of participant assignment to those groups.

Answer: We thank the reviewer for these insightful comments. We agree that the observed differences in beta diversity likely reflect substantial inter-individual variability, which may confound comparisons between sample types across participants. Given this variability, comparisons between groups A and B may indeed be influenced by participant-specific microbiome profiles rather than solely by differences in sampling approach. In light of this, we acknowledge that our initial interpretation may have overstated the distinction between groups based on sampling order. We have therefore revised the manuscript to temper this conclusion and avoid implying the presence of a clear and consistent "vaginal" or "urine" microbiome profile based on these data. Importantly, this also aligns with the broader consideration that FVU is often used as a proxy for the vaginal microbiome rather than a representation of the bladder microbiome. As such, the lack of clear clustering by sample type further supports the notion that FVU captures a mixture of microbial contributions, rather than a distinct niche-specific signal. We have revised the corresponding text to clarify these points and to avoid overinterpretation of the beta diversity analysis (lines 172 – 175). The principal component analysis comparison between groups has also been removed from the revised manuscript.

5. Line 158: Whilst the authors acknowledge some of the limitations of their study, I am convinced that they do not go far enough. First, I understand why they used short read 16S sequencing but they do not acknowledge that it limits identification with confidence at the species level even if they query some of their sequence reads against additional databases - there is just not sufficient sequence data for distinction with confidence between many closely related species. Furthermore, there's no mention of confidence scores for the taxonomic identifications. How confident are the

authors that their identifications are accurate? This is important for the second issue. At the level of their analysis, the authors cannot be certain that the FVU samples truly resemble the vaginal swabs below the level of the most relatively abundant taxa. I stand by my original concern (reproduced below).

Answer: We thank the reviewer for this important point and agree that short-read 16S sequencing has inherent limitation for species-level resolution, mainly among closely related taxa. In our study, taxonomic assignments were therefore not interpreted at the species level. As described in the manuscript (lines 109 – 113), all samples were profiled at the genus level, and for *Lactobacillus* specifically at the subgenus level, following the validated approach used in the Isala study (Lebeer et al. Nat. Microbiology, 2023). We acknowledge that short 16S regions generally do not allow consistent and confident classification to the species level. Accordingly, no species-level abundances are reported or discussed in the current version of the manuscript. We have revised the manuscript to more explicitly acknowledge these limitations and to clarify that conclusions regarding FVU-vaginal similarity are most robust at the (sub)genus level, which is the taxonomic level we assessed using 16S rRNA sequencing (lines 208 – 212).

Regarding classification confidence, taxonomic assignments were performed using the RDP classifier as implemented in the DADA2 R package. This classifier uses a naïve Bayesian approach, estimating confidence scores for each taxonomic rank via bootstrap resampling of the input sequences. Consistent with commonly accepted practice in 16S rRNA gene amplicon studies, a confidence score cutoff of 50/100 was applied for taxonomic assignment. We have clarified this in our methods (cfr. Supplementary Material, lines 57 – 59).

6. "Third, and importantly, the authors base their conclusions on whether the sample composition in terms of alpha (within sample) diversity and/or the identity of the predominant taxon differed. In general, I agree that a simple glance at the data suggests that there is not much difference between the 3 different types of samples for most participants. However, the authors do not determine the likelihood that a taxon detected in the vaginal swabs will also be detected in the FVU or cervicovaginal brush samples. If one only looks at the most relatively abundant taxa, they may miss that important information may be lost by using a proxy sample, such as FVU. The authors should take a look at a recent publication comparing midstream voided urine and vaginal swabs in pregnant females (Sung et al., 2024 PMID: 38088549). These authors asked that very question and found that the two sample types were not particularly good proxies for each other. The authors could use the analytical methods used in this paper to determine whether FVU samples are good proxies for vaginal swabs in this population."

Answer: We thank the reviewer for this important suggestion. We agree that evaluating overall similarity based on diversity metrics or dominant taxa alone may overlook discrepancies in taxon detection between sample types. In response, we have incorporated additional analyses to explicitly quantify concordance at the taxon level between vaginal swabs, first-void urine (FVU), and cervicovaginal brush samples. Specifically, we assessed the probability of detecting a given taxon in one sample type conditional on its presence in another, following an approach comparable to that described by Sung et al. (2024). These analyses provide a more comprehensive evaluation of the suitability of FVU and cervicovaginal brush samples as proxy sampling matrices for vaginal swabs. The

results of these analyses have been incorporated into the revised manuscript (see Results section lines 176 – 189 and Figure 2).

7. Now I understand what the authors are trying to do, and I believe that it is a worthy endeavor, but they MUST include this concern as a limitation. They MUST tell their readers how this simple, non-invasive approach will limit interpretation of the resultant data. Furthermore, the samples represent the urogenital tract and may not be a great proxy for what is actually in the vagina itself. The results will NOT be vaginal microbiome but rather a composite of the entire urogenital microbiome.

Answer: We thank the reviewer for this remark. While the study by Sung et al. (2024) compares midstream urine with vaginal swabs, our study specifically focuses on FVU and cervicovaginal samples as a proxy for the vaginal microbiome rather than the bladder microbiome. Based on our new taxon-level concordance analyses (lines 176 – 189, Figure 2), we found that FVU captures the vaginal microbiome to a considerable extent, supporting its use as a proxy sampling method for vaginal swabs. In addition, we extended these analyses to cervicovaginal brush samples and observed patterns of taxon-level concordance comparable to vaginal swabs. We also acknowledge as a limitation that we did not include paired midstream urine samples collected during the same voiding event and thus cannot preclude that the FVU microbiome is a composite of the urine or bladder microbiome and the vaginal microbiome. We have clarified and emphasized this limitation in the revised manuscript (lines 203 – 206).

Other comments

1. Line 126: Why was this more interesting than the other results? Delete the "interesting." It does nothing positive for the logic flow. Note that these types of transition words usually hold no value as the reader has no idea why an observation was "interesting," "surprising," or "intriguing." If it was interesting, surprising, or intriguing, answer the question "why?" and then use your answer as an transition/introductory phrase.

Answer: We thank the reviewer for this comment and removed the word 'interesting' in line 148 of the revised manuscript.

2. Line 127: "...were predominated by a combination of ..." is a better more accurate way to say what the authors mean.

Answer: We thank the reviewer for this comment and adapted the wording following this suggestion in line 148 of the revised manuscript.

3. Line 138: More accurate is "...less relatively abundant..." because 16S sequencing is semi-quantitative due to several different biases inherent in the method that affect quantification.

Answer: We thank the reviewer for this suggestion and agree that relative abundance is more correct in this context. Thus, we adapted this in line 162 of the revised manuscript.

4. Line 142: Remind the reader how the groups differ. They are described at the very beginning of the Observation, and I had forgotten by the time I got to this sentence.

Answer: We thank the reviewer for this helpful suggestion. However, we have removed this comparison in the revised manuscript, as these differences are likely driven by inter-individual variability rather than the group definitions, as discussed in response to major comment #4. Therefore, we no longer describe these groups in this context.

5. Line 153: Yes. Biases. But the differences between the participants means that you cannot compare them. They have to be their own controls.

Answer: We thank the reviewer for this important remark. We fully agree that, given the high inter-individual variability of the vaginal microbiome, comparisons should be performed within participants, with each individual serving as their own control. Our analyses were indeed conducted using a paired design, focusing on within-subject comparisons between sample types. We have revised the manuscript to clarify this point in the limitations section (lines 192 – 195).

Reviewer #2 (Comments for the Author):

1. Revised manuscript is significantly changed from its original submission. The major change is complete deletion of the qPCR data related to estimating human and bacterial DNA and analyses of that data. This is a pity as such data would have strengthened this pilot study. Authors refer to some sort of 'inconsistencies' in those measurements in their response to several reviewers but it did not provide too much clarify on the issue identified. Unfortunately, the physical samples are stated to be used up and no longer available for re-analyses.

Answer: We thank the reviewer for this thoughtful comment and appreciate the recognition of the potential value of the qPCR data. We agree that, in principle, these measurements could have strengthened the study. However, during the revision process, we identified important limitations that prevent a robust interpretation of these data. Specifically, drawing reliable conclusions would have required measurements of the total DNA concentration prior to PCR amplification, which were unfortunately not included. As the original aliquots are no longer available, it is not possible to perform these additional measurements retrospectively. While we acknowledge this as a limitation, we consider that excluding these data results in a more rigorous and reliable presentation of the findings based on the sequencing analyses performed on the original aliquots.

2. The revised manuscript now has the PCoA analyses for patients and groups. The response file contains the third PCoA analysis for sample storage protocol (Direct freezing or after 7 d RT) - It would be great to include that to the supplemental materials for this manuscript.

Answer: We thank the reviewer for this helpful suggestion. The PCoA analysis for the sample storage protocol (direct freezing versus seven days at room temperature) has been considered. However, we have not included this as a separate panel, as Figure 1d already presents all protocols per participant, thereby encompassing this comparison within the existing visualization.

3. Response file/Abstract/Results and Figure 2 legends differ in interpretation of the presented data on sampling order. Figure 2 legend states that PCoA is nonsignificant for both plots given (by participant and by group) while Abstract/Response file claim clear clusters and differences.

Answer: We thank the reviewer for this valid remark. In the revised version of the manuscript, we decided to leave out the PCoA analyses focusing on differences between groups (based on sampling order). As pointed out by reviewer 1, we agree that we cannot directly compare two groups since our sample size is relatively small. In addition, the vaginal microbiome is known to exhibit considerable inter-individual variability, as shown in Fig. 1d, which further complicates between-group comparisons. Therefore, we decided to focus on within-participant differences in sample type and storage method. This was also mentioned in the limitations section of the revised manuscript (lines 205 – 207).

4. Supplementary Table is not listed in Manuscript's list of Supplementary materials. This table also lacks units or descriptions of values (for example, DNA concentration or Read concentration lack units). Samples in the tables are not sorted for some reason by patient or other cohorts and it is not immediately clear why some participants only have 4 samples instead of 5 (three collection modes with two samples being left out at RT for 7 days).

Answer: We thank the reviewer for this remark. In the revised manuscript we refer to Supplementary Table 1 in line 117, with updated information indicating whether a sample has passed the quality control (QC) process or not, and units for given descriptive values (values for these are only given if the specific sample has passed QC).

5. Observation format is limiting the word numbers for this manuscript and thus is perhaps prohibitive to inclusion of all the details added in the reviewers' comments. Given the importance of the experimental and protocol details for this kind of studies I would suggest including those details into the supplemental materials to avoid any confusions for future readers.

Answer: We thank the reviewer for this valuable suggestion. In line with this recommendation, we have included a new Supplementary Information file that provides a detailed overview of the study design, data collection procedures, sample processing, and analytical workflow. This addition ensures transparency and reproducibility while keeping the main manuscript concise.

6. Additionally, authors did not include any mentioning or acknowledging the urine microbiome or urogenital microbiome as similar or distinct to the vaginal microbiome. It is interesting choice and in particular in regard to recent study mentioned by another reviewer (Sung et al, 2023). Authors refer to the studies from HPV field which seems to be more advanced in developing protocols for viral DNA detection and sampling methods for a few research years. Nevertheless, the human-cell specific virus might not necessarily be a good standard as authors aim to analyze composition of bacteria in those microbial niches and stability of cells and DNA, accumulation of host or microbial cells and so one - might differ. This actually stresses the need for this pilot study but calls for attention to details in experimental descriptions and interpretation of the results.

Answer: We thank the reviewer for this remark. We acknowledge that the urogenital (including urinary) microbiome represents a distinct, yet potentially overlapping ecological niche compared to the vaginal microbiome, and that this distinction is highly relevant to consider when interpreting microbiome profiles from the FVU and cervicovaginal samples.

In contrast to Sung et al. (2024), who compared midstream urine to vaginal swabs, our study focuses on alternative sampling approaches, specifically first-void urine (FVU) and cervicovaginal brushes, in relation to vaginal swabs. Based on our analyses, we observed that both sample types capture key

components of the vaginal microbiome, with FVU showing stronger concordance and representing a promising non-invasive proxy sampling method for vaginal swabs. At the same time, we recognize that FVU may represent a composite signal reflecting contributions from both the vaginal and urinary microbiomes. As we did not include paired midstream urine samples collected during the same voiding event, we cannot fully disentangle these contributions. This limitation has now been clearly acknowledged and emphasized in the revised manuscript (lines 203 – 205).

Re: Spectrum00096-26R1 (**First-void urine and cervicovaginal brushes for vaginal microbiome profiling: a proof-of-concept study**)

Dear Prof. Sarah Lebeer:

Your manuscript has been accepted, and I am forwarding it to the ASM production staff for publication. Your paper will first be checked to make sure all elements meet the technical requirements. ASM staff will contact you if anything needs to be revised before copyediting and production can begin. Otherwise, you will be notified when your proofs are ready to be viewed.

Data Availability: ASM policy requires that data be available to the public upon online posting of the article, so please verify all links to sequence records, if present, and make sure that each number retrieves the full record of the data. If a new accession number is not linked or a link is broken, provide production staff with the correct URL for the record. If the accession numbers for new data are not publicly accessible before the expected online posting of the article, publication may be delayed; please contact ASM production staff immediately with the expected release date.

Publication Fees: For information on publication fees and which article types have charges, please visit our website. We have partnered with Copyright Clearance Center (CCC) to collect author charges. If fees apply to your paper, you will receive a message from no-reply@copyright.com with further instructions. For questions related to paying charges through RightsLink, please contact CCC at ASM_Support@copyright.com or toll free at +1-877-622-5543. CCC makes every attempt to respond to all emails within 24 hours.

ASM Membership: Corresponding authors may join or renew ASM membership to obtain discounts on publication fees. Need to upgrade your membership level? Please contact Customer Service at Service@asmusa.org.

Embargo Policy: A press release may be issued as soon as the manuscript is posted on the Spectrum Latest Articles webpage. The corresponding author will receive an email with the subject line "ASM Journals Author Services Notification" when the article is available online.

Cover Image Submissions: If you would like to submit a potential Cover Image, please email a file and a short legend to spectrum@asmusa.org. Please note that we can only consider images that (i) the authors created or own and (ii) have not been previously published. By submitting, you agree that the image can be used under the same terms as the published article. Image File requirements: TIF/EPS, 7.5 inches wide by 8.25 inches tall (at least 2,250 pixels wide by 2,475 pixels tall), minimum 300 dpi resolution (600 dpi preferred), RGB, and no figure elements, e.g., arrows or panel labels. The legend should be a short description of the image, 1-2 sentences recommended. Please download and use this interactive template in Adobe the https://journals.asm.org/pb-assets/html/asm-cover-sizing-tool.html to ensure that your proposed cover image meets our size requirements (https://journals.asm.org/pb-assets/pdf-text-excel-files/ASM-Interactive-Sizing-Cover-Template-1715689791.pdf).

The ASM Journals program strives for constant improvement in our submission and publication process. Please tell us how we can improve your experience by taking this quick Author Survey.

Thank you for submitting your paper to Spectrum.

Sincerely,
Zhe Li
Editor
Microbiology Spectrum

Reviewer #1 (Comments for the Author):

The authors have responded positively to the previous review. The result is a much better more accurate description of their work.
