# Supplementary material for: First-void urine and cervicovaginal brushes for vaginal microbiome profiling: a proof-of-concept study
Source: Microbiol Spectr. 2026 Aug 19;14(10):e00096-26. doi: 10.1128/spectrum.00096-26 (PMC13689475; doi:10.1128/spectrum.00096-26)
Supplement: Supplementary file 2 [file spectrum.00096-26-s0003.docx]

**Supplementary Material**

**First-void urine and cervicovaginal brushes for vaginal microbiome profiling: a proof-of-concept study**

Maria Pinedo-Bardales^1,*^, Anne Van Caesbroeck^2,*^, Sarah Ahannach^1,3^, Margo Bell^2^, Severien Van Keer^2^, Laura Téblick^2^, Isabel Erreygers^1^, Sam Bakelants^1^, Sarah Van den Bosch^1^, Sandra Condori-Catachura^1^, Stijn Wittouck^1^, Alex Vorsters^2,$^, Sarah Lebeer^1,3,$,°^

^1^Laboratory of Applied Microbiology and Biotechnology, Department of Bioscience Engineering, University of Antwerp, Antwerp, Belgium

^2^Centre for the Evaluation of Vaccination (CEV), Vaccine & Infectious Disease Institute (VAXINFECTIO), Faculty of Medicine and Health Sciences, University of Antwerp, Antwerp, Belgium

^3^U-MaMi Centre of Excellence, University of Antwerp, Antwerp, Belgium

* shared first authors

$ shared senior authors

° corresponding author

Correspondence: [sarah.lebeer@uantwerpen.be](mailto:sarah.lebeer@uantwerpen.be)

**Data collection, processing and analytical workflow**

**Ethics approval**

This study was approved by the Institutional Review Board (Ethics Committee) of Antwerp University Hospital/University of Antwerp (B300201734129) and conducted according to the guidelines of the Declaration of Helsinki. Written informed consent was obtained from all participants before study enrolment.

**Participants recruitment and sample collection**

Self-reported healthy, reproductive-age women (n=10) were recruited at the Centre for the Evaluation of Vaccination of the University of Antwerp (Antwerp, Belgium) in October 2023. Sample collection was conducted in two groups of five participants, each following a different sampling order (Fig. S1). All samples were self-collected.

Group A participants first collected a FVU sample using the 20 mL Colli-Pee device (Novosanis Colli-Pee, part of the Orasure Technologies Inc. Group, Pennsylvania, USA), which was prefilled with 1:3 urine conservation medium (UCM) (DNA Genotek Inc, Ottawa, Canada). Participants were instructed not to wash their genitals thoroughly and to avoid urinating for at least 1 hour before sample collection. This was followed by the collection of two vaginal samples using eNAT swabs (Copan, Brescia, Italy) in a vial prefilled with 1 mL of lysis buffer, and a cervicovaginal sample using the Evalyn Brush (Rovers Medical Services B.V., Oss, The Netherlands).

In Group B, participants collected samples using the same devices, but in a different order: first the FVU sample, followed by the cervicovaginal brush and then two vaginal swabs. The Evalyn Brushes were resuspended in 20 mL of ThinPrep PreservCyt solution (Hologic, Massachusetts, USA). All samples were aliquoted in 1 mL Eppendorf tubes after thorough vortexing for 15-30 seconds. One of each participant’s vaginal swabs, and one 1 mL aliquot of FVU were kept at RT for up to seven days and subsequently frozen at -20°C. All other aliquots were immediately frozen at -20°C. Samples were stored at Biobank Antwerp (Antwerp, Belgium, ID: BE 71030031000).

**DNA extraction and 16S rDNA amplicon sequencing**

DNA was extracted from all 1 mL aliquots using the DNeasy PowerSoil Pro Kit (Qiagen, Germany) with the Qiacube connect robot (Qiagen, Germany) following the procedure described by Ahannach *et al*.^1^. The V4 region of the 16S rRNA gene of each bacterial DNA was amplified using standard primers altered for dual-index pair-end sequencing^2^. Library preparation was performed using standard in-house conditions^3^. Dual-index paired-end sequencing was performed on a MiSeq Desktop sequencer (Illumina). The sequencing run encompassed all DNA samples alongside buffer controls (UCM and ThinPrep PreservCyt solution) and two PCR-grade water controls to ensure validity of the DNA extraction processes and polymerase chain reaction (PCR).

**Processing and quality control of 16S sequencing data**

Quality control and processing of amplicon reads were performed with the R package DADA2 version 1.22.0^4^ following previously established pipelines detailed by Lebeer *et al.*^3^. The merged and denoised reads (amplicon sequence variants, ASVs) underwent taxonomic annotation from the phylum to the genus level using the assignTaxonomy function and the Genome Taxonomy Database (GTDB)^5^ ssu archive (version 207), applying a confidence cutoff of 50/100.

To enhance taxonomic resolution specifically for the genus *Lactobacillus*, ASVs from this genus were reclassified into nine different subgenera validated previously in vaginal samples from the Isala cohort^3^. ASVs from the genus *Bifidobacterium* in GTDB were reclassified using the SILVA database (version 138)^6^.

Taxon and sample quality control measures were applied as follows. ASVs identified as non-bacterial, including mitochondria, and those exceeding 260 bases in length were excluded. Samples were manually filtered based on read concentration (total read count divided by pooled volume) and any sample with a value lower than the maximum observed in the negative control was removed.

**Microbiome profiling and assessment of concordance between sampling methods**

Taxonomic profiles of high-quality samples and diversity metrics were visualized using the in-house developed R package tidytacos (<https://github.com/LebeerLab/tidytacos>)^7^. Alpha diversity was assessed using the inverse Simpson index, and beta diversity using the Bray-Curtis dissimilarity index (BC). Additional analyses included the calculation of BCs based on taxa presence/absence and Principal Coordinates Analysis (PCoA) for clustering. In our analysis, we used the vegan package implementation of Bray-Curtis dissimilarity within the tidytacos workflow, which allows calculation on both quantitative (abundance) data and binary (presence/absence) data. Specifically, for the presence/absence-based analyses, the count table was first transformed to a binary format prior to distance calculation. When Bray-Curtis is applied to such binarized data, it effectively represents a qualitative dissimilarity measure that reflects shared versus non-shared taxa and is mathematically equivalent to the Sørensen (Dice) index. Statistical comparisons between sampling devices and protocols were performed using the Wilcoxon signed-rank test with Holm-Bonferroni correction.

Taxon-level concordance between sample types was assessed following the approach of Sung et al.^8^ Briefly, microbiome profiles were converted to relative abundance and taxa were considered present above a threshold of 0.5%. Presence-absence matrices were generated for paired directly frozen samples (vaginal swabs vs. FVU samples and vaginal swabs vs. cervicovaginal brushes samples) within individuals, and rare taxa (<5 detections considering both sample types) were excluded. Conditional probabilities were then calculated to quantify the likelihood of detecting a taxon in one sample type given its presence in the other, providing a measure of concordance between sampling methods. These probabilities were visualized using heatmaps generated in R Studio, facilitating intuitive comparison of taxon-level agreement between sample types.

**References**

1.	Ahannach, S. *et al.* Microbial enrichment and storage for metagenomics of vaginal, skin, and saliva samples. *iScience* **24**, 103306 (2021).

2.	Kozich, J. J., Westcott, S. L., Baxter, N. T., Highlander, S. K. & Schloss, P. D. Development of a dual-index sequencing strategy and curation pipeline for analyzing amplicon sequence data on the MiSeq Illumina sequencing platform. *Appl. Environ. Microbiol.* **79**, 5112–5120 (2013).

3.	Lebeer, S. *et al.* A citizen-science-enabled catalogue of the vaginal microbiome and associated factors. *Nat. Microbiol.* **8**, 2183–2195 (2023).

4.	Callahan, B. J. *et al.* DADA2: High-resolution sample inference from Illumina amplicon data. *Nat. Methods* **13**, 581–583 (2016).

5.	Parks, D. H. *et al.* GTDB: an ongoing census of bacterial and archaeal diversity through a phylogenetically consistent, rank normalized and complete genome-based taxonomy. *Nucleic Acids Res.* **50**, D785–D794 (2022).

6.	Quast, C. *et al.* The SILVA ribosomal RNA gene database project: improved data processing and web-based tools. *Nucleic Acids Res.* **41**, D590–D596 (2013).

7.	Wittouck, S., Rillaer, T. V., Smets, W. & Lebeer, S. Tidytacos: An R package for analyses on taxonomic composition of microbial communities. *J. Open Source Softw.* **10**, 6313 (2025).

8.	Sung, J. *et al.* First trimester “clean catch” urine and vaginal swab sample distinct microbiological niches. *Microbiol. Spectr.* **12**, e02638-23 (2023).

**Supplementary Figures**

**Figure S1: Overview of the study workflow.** Sample collection: sampling sequence for group A (*n* = 5, participants 01, 02, 03, 04, and 05) was first-void urine (FVU, collected using Colli-Pee®) followed by two vaginal swabs (eNAT® and a cervicovaginal brush (Evalyn® Brush). Sampling sequence for group B (*n* = 5, participants 06, 07, 08, 09, and 10) was FVU, followed by a cervicovaginal brush and two vaginal swabs. Sample processing: a 1 mL aliquot of the 20 mL FVU and cervicovaginal brush samples was used. Two storage conditions were compared for FVU samples and vaginal swabs: seven days at room temperature (7d RT) or directly frozen at -20°C (direct). DNA extraction: DNA was extracted from 200 µL of sample using the DNeasy PowerSoil Pro kit (Qiagen) using automatic DNA extraction using the Qiacube connect robot. Sequencing: Dual index pair-end sequencing of the V4 region of the 16S rRNA gene using a Miseq sequencer (Ilumina). Statistical analysis included taxonomic annotation, Principal Coordinates Analysis and statistical testing using the Wilcoxon signed-rank test with Holm-Bonferroni corrected p-values.

**Figure S2: Quality control of the samples based on the negative controls.** The sequencing run encompasses DNA extracts originating from all samples and controls. Read concentration and read count for both the samples and controls are presented in (a) with the blue line representing the threshold of read concentration, while the taxonomic composition of the controls is shown in (b). NC_ucm = negative control (NC) of the urine conservation medium (UCM). NC_prep = NC of the ThinPrep PreservCyt solution. NC_dna = DNA extraction control (PCR-grade water). NC_pcr = PCR cleanup controls (PCR-grade water). eNAT direct = vaginal swab collected using the eNAT® system and directly frozen; eNAT 7d RT = vaginal swab collected using the eNAT® system and kept for seven days at room temperature (RT); FVU = first-void urine samples collected using a 20 mL Colli-Pee device and directly frozen; FVU 7d RT = first-void urine samples collected using a 20 mL Colli-Pee device and kept for seven days at room temperature. Evalyn direct = cervicovaginal sample collected using Evalyn Brush and directly frozen.

**Supplementary Tables**

Supplementary Table 1: Overview of samples included in this study. ID: participant number; NA: not applicable (the sample did not pass quality control); QC: quality control; RT: room temperature; Group: in group A, participants collected the FVU sample first, followed by two vaginal swabs, and finishing with the Evalyn brush. In group B, participants collected samples using the same devices, but in a different order: first, the FVU sample, followed by the cervicovaginal brush and then two vaginal swabs.

| **ID** | **Group** | **Sampling device** | **Storage condition** | **QC passed** | **Total bacterial reads** | **Predominant taxa*** | **Read concentration (read counts/µL)** |
| --- | --- | --- | --- | --- | --- | --- | --- |
| 1 | A | eNAT | Frozen immediately | Yes | 16272 | *Lactobacillus gasseri* group and *Gardnerella* | 13343.04 |
| 1 | A | eNAT | Stored for 7d at RT | Yes | 14308 | *Lactobacillus gasseri* group and *Gardnerella* | 16382.66 |
| 1 | A | Colli-Pee | Frozen immediately | Yes | 16094 | *Gardnerella* | 7371.05 |
| 1 | A | Colli-Pee | Stored for 7d at RT | Yes | 19103 | *Gardnerella* | 12512.47 |
| 1 | A | Evalyn brush | Frozen immediately | No | NA | NA | NA |
| 2 | A | Colli-Pee | Frozen immediately | Yes | 21577 | *Lactobacillus crispatus* group | 24532.28 |
| 2 | A | eNAT | Frozen immediately | Yes | 23035 | *Lactobacillus crispatus* group | 31677.04 |
| 2 | A | eNAT | Stored for 7d at RT | Yes | 25546 | *Enterobacter* | 9774.38 |
| 2 | A | Colli-Pee | Stored for 7d at RT | Yes | 25802 | *Enterococcus* | 16771.3 |
| 2 | A | Evalyn brush | Frozen immediately | No | NA | NA | NA |
| 3 | A | Colli-Pee | Frozen immediately | Yes | 17355 | No dominance | 14286.86 |
| 3 | A | Colli-Pee | Stored for 7d at RT | Yes | 22979 | No dominance | 13164.69 |
| 3 | A | eNAT | Frozen immediately | Yes | 17423 | No dominance | 10846.88 |
| 3 | A | eNAT | Stored for 7d at RT | Yes | 17097 | No dominance | 17693.83 |
| 3 | A | Evalyn brush | Frozen immediately | Yes | 17530 | *Gardnerella* | 9203.25 |
| 4 | A | eNAT | Frozen immediately | Yes | 14894 | *Lactobacillus gasseri* group and *Gardnerella* | 8787.46 |
| 4 | A | eNAT | Stored for 7d at RT | Yes | 14109 | *Lactobacillus gasseri* group and *Gardnerella* | 15660.99 |
| 4 | A | Colli-Pee | Stored for 7d at RT | No | NA | NA | NA |
| 4 | A | Colli-Pee | Frozen immediately | Yes | 16803 | *Lactobacillus gasseri* group and *Gardnerella* | 6359.94 |
| 4 | A | Evalyn brush | Frozen immediately | Yes | 4723 | *Gardnerella* | 1513.72 |
| 5 | A | eNAT | Frozen immediately | Yes | 19491 | *Lactobacillus iners* group | 15982.62 |
| 5 | A | eNAT | Stored for 7d at RT | Yes | 21801 | *Lactobacillus iners* group | 21146.97 |
| 5 | A | Colli-Pee | Frozen immediately | Yes | 21765 | *Lactobacillus iners* group | 12514.87 |
| 5 | A | Colli-Pee | Stored for 7d at RT | Yes | 57841 | *Enterococcus* | 53792.13 |
| 5 | A | Evalyn brush | Frozen immediately | No | NA | NA | NA |
| 6 | B | eNAT | Frozen immediately | Yes | 25574 | *Lactobacillus crispatus* group | 17518.19 |
| 6 | B | eNAT | Stored for 7d at RT | Yes | 19910 | *Lactobacillus crispatus* group | 18516.3 |
| 6 | B | Colli-Pee | Frozen immediately | Yes | 22702 | *Lactobacillus crispatus* group | 12145.57 |
| 6 | B | Colli-Pee | Stored for 7d at RT | No | NA | NA | NA |
| 6 | B | Evalyn brush | Frozen immediately | Yes | 11016 | *Lactobacillus crispatus* group | 3409.45 |
| 7 | B | eNAT | Frozen immediately | Yes | 22789 | *Lactobacillus crispatus* group | 20168.26 |
| 7 | B | eNAT | Stored for 7d at RT | Yes | 21654 | *Lactobacillus crispatus* group | 24252.48 |
| 7 | B | Colli-Pee | Frozen immediately | Yes | 21789 | *Lactobacillus iners* group | 6209.86 |
| 7 | B | Colli-Pee | Stored for 7d at RT | Yes | 27974 | *Lactobacillus iners* group and *Lactobacillus crispatus* group | 10420.32 |
| 7 | B | Evalyn brush | Frozen immediately | Yes | 9828 | *Lactobacillus crispatus* group | 4963.14 |
| 8 | B | eNAT | Frozen immediately | Yes | 12582 | *Lactobacillus jensenii* group | 6209.22 |
| 8 | B | eNAT | Stored for 7d at RT | Yes | 10462 | *Lactobacillus jensenii* group | 5754.1 |
| 8 | B | Colli-Pee | Frozen immediately | Yes | 15915 | *Lactobacillus jensenii* group | 6946.9 |
| 8 | B | Colli-Pee | Stored for 7d at RT | No | NA | NA | NA |
| 8 | B | Evalyn brush | Frozen immediately | Yes | 4405 | *Lactobacillus jensenii* group | 2202.5 |
| 9 | B | eNAT | Frozen immediately | Yes | 18947 | *Lactobacillus iners* group | 14778.66 |
| 9 | B | eNAT | Stored for 7d at RT | Yes | 24764 | *Lactobacillus iners* group | 23897.26 |
| 9 | B | Colli-Pee | Frozen immediately | Yes | 27612 | *Lactobacillus iners* group | 13806 |
| 9 | B | Colli-Pee | Stored for 7d at RT | Yes | 32544 | *Enterobacter* | 24733.44 |
| 9 | B | Evalyn brush | Frozen immediately | Yes | 10041 | *Lactobacillus crispatus* group | 4352.77 |
| 10 | B | eNAT | Frozen immediately | Yes | 22157 | *Lactobacillus crispatus* group | 8585.84 |
| 10 | B | eNAT | Stored for 7d at RT | Yes | 23507 | *Lactobacillus crispatus* group | 23389.46 |
| 10 | B | Colli-Pee | Frozen immediately | No | NA | NA | NA |
| 10 | B | Colli-Pee | Stored for 7d at RT | No | NA | NA | NA |
| 10 | B | Evalyn brush | Frozen immediately | Yes | 1237 | *Lactobacillus crispatus* group | 364.92 |
